# Long-Time Prediction of Arrhythmic Cardiac Action Potentials Using Recurrent Neural Networks and Reservoir Computing

**DOI:** 10.3389/fphys.2021.734178

**Published:** 2021-09-27

**Authors:** Shahrokh Shahi, Christopher D. Marcotte, Conner J. Herndon, Flavio H. Fenton, Yohannes Shiferaw, Elizabeth M. Cherry

**Affiliations:** ^1^School of Computational Science and Engineering, Georgia Institute of Technology, Atlanta, GA, United States; ^2^School of Physics, Georgia Institute of Technology, Atlanta, GA, United States; ^3^Department of Physics & Astronomy, California State University, Northridge, CA, United States

**Keywords:** reservoir computing, recurrent neural network, echo state network, time series forecasting, cardiac action potential

## Abstract

The electrical signals triggering the heart's contraction are governed by non-linear processes that can produce complex irregular activity, especially during or preceding the onset of cardiac arrhythmias. Forecasts of cardiac voltage time series in such conditions could allow new opportunities for intervention and control but would require efficient computation of highly accurate predictions. Although machine-learning (ML) approaches hold promise for delivering such results, non-linear time-series forecasting poses significant challenges. In this manuscript, we study the performance of two recurrent neural network (RNN) approaches along with echo state networks (ESNs) from the reservoir computing (RC) paradigm in predicting cardiac voltage data in terms of accuracy, efficiency, and robustness. We show that these ML time-series prediction methods can forecast synthetic and experimental cardiac action potentials for at least 15–20 beats with a high degree of accuracy, with ESNs typically two orders of magnitude faster than RNN approaches for the same network size.

## 1. Introduction

Cardiac electrical signals, known as action potentials, exhibit complex non-linear dynamics, including period-doubling bifurcations in their duration (Guevara et al., 1984; Watanabe et al., 2001) and amplitude (Chen et al., 2017), along with higher-order period-doublings (Gizzi et al., 2013) and chaotic behavior (Chialvo et al., 1990). Potentially life-threatening states like fibrillation often are preceded by such long-short oscillations in action potential duration or amplitude known as alternans in the medical literature (Nolasco and Dahlen, 1968; Pastore et al., 1999; Gizzi et al., 2013; Chen et al., 2017). A number of methods for control of cardiac alternans have been developed (Rappel et al., 1999; Christini et al., 2006; Berger et al., 2007; Garzón et al., 2009; Garzon et al., 2014; Kulkarni et al., 2018), and while some have been demonstrated in cardiac experimental preparations (Christini et al., 2006; Kulkarni et al., 2018), they have not yet found clinical application in part because of the limited length scales over which control can be accomplished (Echebarria and Karma, 2002; Garzon et al., 2014; Otani, 2017). An alternative strategy focusing on preventing rather than controlling alternans could be more attractive clinically, but such an approach would require accurate prediction of when such dynamics would occur.

Data-driven approaches can be used to forecast systems like cardiac action potentials by inferring the dynamics from observed data represented as time series (Kutz, 2013). Along with conventional techniques for time-series modeling and forecasting like autoregression approaches (Stock and Watson, 2001; Ing, 2003) and dynamic mode decomposition (Schmid, 2010), machine-learning methods have become increasingly used for predicting dynamical system states (Kutz, 2013; Chattopadhyay et al., 2020; Dubois et al., 2020). Recent years have seen significant advances in the field of machine learning, especially deep learning techniques. Recurrent neural networks (RNNs) have been successfully employed in dynamical domains, as the recurrent connections in the network provide a notion of memory and allow them to naturally embed temporal information. However, RNNs are still trained using the computationally expensive technique of back-propagation through time and remain prone to vanishing and exploding gradient problems. Gated RNNs can help overcome some of these problems; for example, to overcome the vanishing gradient problem, gated RNNs take advantage of memory cell architecture and a gating mechanism allowing the network to select which information should be kept and which forgotten (Hochreiter and Schmidhuber, 1997). This process enables the network to learn the long-term dependencies in sequential temporal data. Two widely used gated RNN approaches include long short-term memory (LSTM) networks and gated recurrent units (GRUs).

An alternative approach for modeling and predicting dynamical systems is reservoir computing (RC) (Lukoševičius and Jaeger, 2009; Sun et al., 2020), where, in contrast to other RNN architectures, the training remains limited to the output layer and the remaining parameters are selected randomly. Despite this simplification compared to other RNN architectures, RC techniques, including the commonly used echo state network (ESN) approach (Jaeger, 2002; Lukoševičius, 2012), have been used successfully to provide accurate multi-step-ahead predictions in non-linear and chaotic time series with very low computational costs (Bianchi et al., 2017; Han et al., 2021). Variations of ESNs, including clustered ESNs, where the reservoir consists of multiple sparsely connected sub-reservoirs (Deng and Zhang, 2006; Junior et al., 2020), and hybrid ESNs, which include input from a mathematical model and are a type of physics-informed machine learning technique (Oh, 2020; Willard et al., 2020), have been shown to have good performance in some cases (Pathak et al., 2018; Doan et al., 2019).

In this work, we show that it is possible to accurately predict future sequences of cardiac action potentials from complex voltage activity obtained *in silico* and in *ex-vivo* experiments. We further compare the performance of several machine-learning techniques for a multi-step prediction of complex cardiac action potential time series. In particular, we consider the accuracy and computational efficiency of LSTMs and GRUs along with ESNs, including a clustered architecture and a physics-informed hybrid option, for different network sizes.

## 2. Methods

Below we provide a brief overview of machine-learning-based time series forecasting methods, describe the datasets we use, and give the details of our specific implementations.

### 2.1. Time Series Forecasting Methods

In this section, we provide a brief summary of the machine-learning approaches we use to forecast cardiac action potential time series.

#### 2.1.1. Gated Recurrent Neural Networks

Recurrent neural networks (RNN) were introduced as a special class of neural networks in which the recurrent connections allow information to persist in the network. However, they suffer from vanishing and exploding gradient problems, which limit their ability to learn long-term dependencies in temporal sequences. Gated RNNs like long short-term memory networks (LSTMs) were developed to remedy such problems. These networks employ memory cells and a gating mechanism to address exactly these issues. Supplementary Figure 1A illustrates the information flow in an LSTM cell. In an LSTM network, a hidden state *h*_*t*_ is calculated using a map formalism:


(1) $$\begin{array}{l} \begin{array}{l} i_{t}=\sigma \left(W_{i}x_{t}+U_{i}h_{t-1}+b_{i}\right),\\ f_{t}=\sigma \left(W_{f}x_{t}+U_{f}h_{t-1}+b_{f}\right),\\ o_{t}=\sigma \left(W_{o}x_{t}+U_{o}h_{t-1}+b_{o}\right),\\ \tilde{c_{t}}=\tanh\left(W_{c}x_{t}+U_{c}h_{t-1}+b_{c}\right),\\ c_{t}=f_{t}⊙c_{t-1}+i_{t}⊙\tilde{c_{t}},\\ h_{t}=\tanh\left(c_{t}\right)⊙o_{t}, \end{array} \end{array}$$

where *i*_*t*_, *f*_*t*_, and *o*_*t*_ denote the input, forget, and output gates, at time *t*, respectively; *x*_*t*_ is the input vector; *W* and *U* are the weight matrices that along with biases *b* are adjusted during the learning process, *c*_*t*_ is the cell state (the internal memory of the LSTM unit), and $\tilde{c_{t}}$ is the cell input activation vector. In these equations, each σ function is sigmoidal and ⊙ denotes Hadamard element-wise multiplication.

Gated recurrent units (GRUs) also were introduced to avoid vanishing and exploding gradient problems and share many similarities in architecture and performance with LSTM networks. The GRU memory cell can be considered as a simplification of an LSTM cell (see Supplementary Figure 1B). Compared to an LSTM memory cell, in a GRU unit, the input and forget gates are combined into a single update gate. This simplification considerably reduces the number of trainable weights and makes GRUs more computationally efficient; at the same time, the prediction does not experience a considerable deterioration in most cases and in some applications may even improve (Bianchi et al., 2017). The GRU equations are given by


(2) $$\begin{array}{l} \begin{array}{l} z_{t}=\sigma \left(W_{z}x_{t}+U_{z}h_{t-1}+b_{z}\right),\\ r_{t}=\sigma \left(W_{r}x_{t}+U_{r}h_{t-1}+b_{r}\right),\\ \tilde{h_{t}}=\tanh\left(W_{h}x_{t}+U_{h}\left(r_{t}⊙h_{t-1}\right)+b_{h}\right),\\ h_{t}=\left(1-z_{t}\right)⊙h_{t-1}+z_{t}⊙\tilde{h_{t}}, \end{array} \end{array}$$

where *z*_*t*_ and *r*_*t*_ are update and reset gates, respectively, and $\tilde{h_{t}}$ is the candidate state. During the training process, the weight matrices *W* and *U* and the bias vector *b* are adjusted, thereby enabling the update and reset gates to select which information should be kept through time and which information is irrelevant for the problem and can be forgotten.

#### 2.1.2. Echo State Networks

ESNs are a simple yet successful RNN architecture in which most of the network parameters are initialized randomly and remain untrained. Supplementary Figures 2A,B demonstrates the main components of an ESN. The hidden layer in an ESN is called the reservoir, which is a randomly initialized RNN.

The reservoir state *h*_*t*_ is updated according to


(3) $$\begin{array}{l} h_{t}=\left(1-\alpha \right)h_{t-1}+\alpha \tanh\left(W^{in}x_{t}+Wh_{t-1}\right), \end{array}$$

where *W*^*in*^ and *W* are the input weight and reservoir weight matrices, respectively; both are initialized randomly and remained untrained. We utilize an extension of the standard ESN formalism that includes a “leaky” update model, which explicitly includes a linear history term. The input signal is denoted by *x*_*t*_ and the constant parameter α ∈ [0, 1] is known as the leaking rate. The output of the network is calculated by the following equation:


(4) $$\begin{array}{l} y_{t}=f^{out}\left(W^{out}\left[x_{t};h_{t}\right]\right), \end{array}$$

where *f*^*out*^ is the output layer activation function, which is chosen here as a unity function. The output weights *W*^*out*^ are obtained here by regularized least-square regression with Tikhonov regularization to avoid overfitting.

Since the initial success of reservoir computing techniques and ESNs, a variety of network topologies have been proposed in the literature, including clustered reservoirs and deep ESNs. The main components of the network are similar to the baseline ESN architecture except for the reservoir topology; for clustered ESNs, the randomized connections between neurons form a set of sub-reservoirs sparsely connected to each other. The network topology is schematically illustrated in Supplementary Figures 2C,D and the update and training equations are the same as those for the baseline ESN (Equations 3 and 4).

We also consider a hybrid ESN approach, which is a physics-informed machine learning approach in which a knowledge-based model is integrated into an ESN; the model and ESN operate simultaneously during the training and prediction. The architecture of this approach is presented in Supplementary Figure 2E. For our application, the network in this design is driven with three input signals: *u*_1_(*t*), the pacing stimulus exciting the network at prescribed intervals; *u*_2_(*t*) = *V*_KB_(*t*), the knowledge-based model providing the voltage dynamics of a cardiac cell; and *u*_3_(*t*) = *V*(*t*), the synthetic or experimental voltage measurements. The knowledge-based model can be a much simpler (typically imperfect) model that provides an approximation of the dynamical behavior of the system, such as the two-variable Mitchell-Schaeffer (Mitchell and Schaeffer, 2003) or three-variable Fenton-Karma (Fenton and Karma, 1998) model, to increase the predictive ability of the network. Consequently, the time evolution of the reservoir state *h*_*t*_ is given by the same Equation 3, where the input signal vector is formed as follow,


(5) $$x_{t}=\left[\left(u_{1}(t);u_{2}(t);u_{3}(t\right)\right].$$

Here we use the Corrado-Niederer update of the Mitchell-Schaeffer model (Corrado and Niederer, 2016) with τ_*in*_ = 0.3 ms, τ_*out*_ = 6 ms, τ_*open*_ = 120 ms, τ_*close*_ = 150 ms, and *v*_*gate*_ = 0.13.

### 2.2. Datasets

To evaluate and compare the performance of these approaches in forecasting cardiac action potential time series, the methods are applied to two synthetic datasets derived from cardiac cell models and to an experimental dataset. We describe the three datasets used below.

#### 2.2.1. Fenton-Karma Model-Derived Dataset

As one dataset, we use a time series of randomly timed action potentials generated using the Fenton-Karma (FK) model (Fenton and Karma, 1998), which includes a voltage variable and two gating variables. The model uses the Beeler-Reuter fitting of the FK model (parameter set 3 in Fenton et al., 2002) and is paced with cycle lengths drawn from a normal distribution using a 2-ms square stimulus current with magnitude 0.4 for 100 beats. To ensure a wide range of action potential durations, the cycle length distribution is centered at 320 ms with a standard deviation of 50 ms. The differential equations of the model are solved using the forward Euler method with a fixed time step of 0.1 ms; this time series is coarsened to obtain the synthetic voltage dataset (see section 2.3.4). The voltage data is then coupled with the stimulus timing so that a multivariate dataset is used, as explained in section 2.3.5.

**Figure 1:**
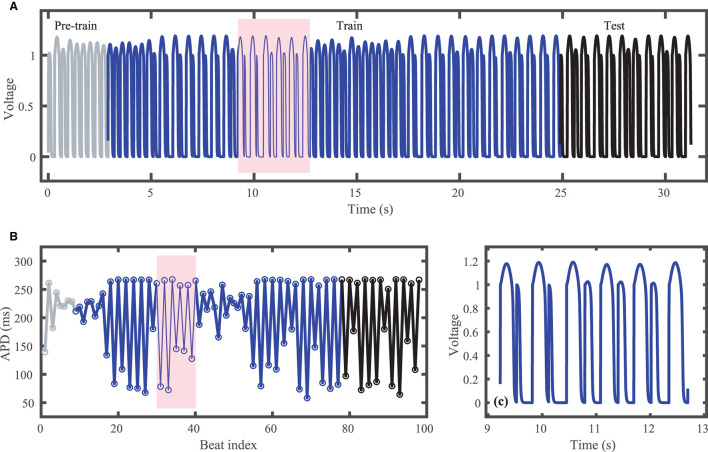
Synthetic action potential time series generated by the Fenton-Karma (FK) model with randomly distributed cycle lengths. **(A)** Voltage time series including unused pre-training data (gray), training data (blue), and testing (prediction) data (black). **(B)** Corresponding APDs, following the same color scheme as **(A)**. **(C)** Zoomed in voltage trace corresponding to the shaded region in **(A,B)** showing the irregular action potential shapes and durations.

Figure 1A shows the voltage trace that together with the corresponding stimulus input form the FK dataset; Figure 1B shows the corresponding action potential duration (APD) values. Data selected for training are shown in blue and testing data are shown in black. Just over 80 action potentials are used for training and about 20 for testing. Because the cycle lengths used include values both above and below the bifurcation to alternans, the resulting APDs included in the dataset span a range of about 200 ms. Figure 1C shows a blowup of the shaded regions in Figures 1A,B to illustrate the irregular timing of stimuli and variation in voltage responses within the training data.

#### 2.2.2. Noble Model-Derived Dataset

As a different type of model-derived dataset, we use the four-variable Noble model (Noble, 1962) for a Purkinje cell in the absence of external pacing. To provide variation in action potential timing and duration, the Noble model is coupled to the three-variable Lorenz model (Lorenz, 1963) in the chaotic regime (ρ = 28, *b* = 8/3, and σ = 10). Time is effectively rescaled in the Lorenz system by multiplying each of the three differential equations by a factor of 0.001. The anionic current conductance in the Noble model was set to be proportional to the *z* variable of the Lorenz model, thereby driving oscillations in the anionic current magnitude in concert with the Lorenz oscillations. Specifically, the conductance was set to 0 for *z* = 0 and to 0.2 for *z* = 60. This extension provides two important features of this dataset: first, the variation in cycle lengths is driven by a chaotic, rather than a random process, and second, there is no need for application of an external stimulus, as action potentials occur when the cell is quiescent and the Lorenz-driven current brings the voltage above the threshold for excitation. Therefore, in this case, only a univariate time series of voltage data is provided, with no external stimulus data. All other model parameters remain as specified in Noble (1962).

**Figure 2:**
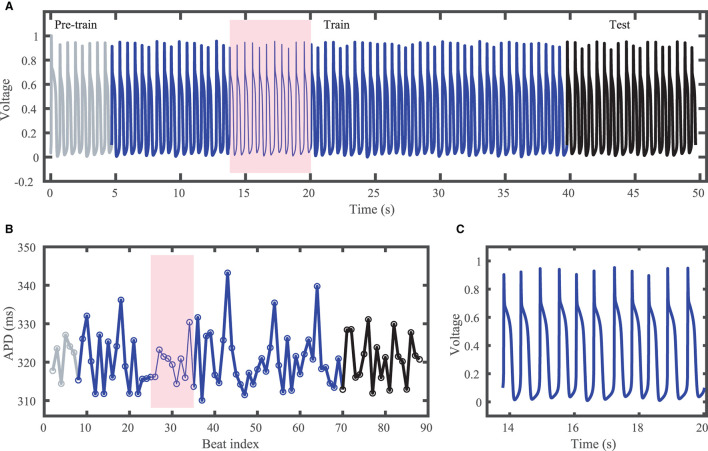
Synthetic action potential time series generated by the Noble model driven by the chaotic Lorenz model. **(A)** Voltage time series including unused pre-training data (gray), training data (blue), and testing (prediction) data (black). **(B)** Corresponding APDs, following the same color scheme as **(A)**. **(C)** Zoomed in voltage trace corresponding to the shaded region in **(A,B)** showing the irregular action potential shapes and durations.

The Noble dataset voltage trace and action potentials are shown in Figure 2, with the training portion (around 65 action potentials) shown in blue and the testing portion (14 action potentials) in black. Because of the inclusion of the chaotic Lorenz model as a driving force, the Noble model-derived dataset demonstrates considerable variation in action potentials, with no consistent pattern. APDs vary between about 310 and 345 ms.

#### 2.2.3. Experimental Dataset

**Figure 3:**
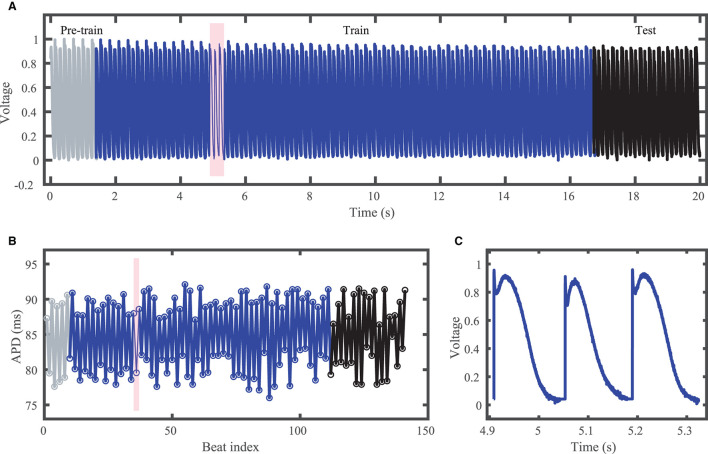
Experimental zebrafish action potential time series featuring irregular alternans characterized by action potentials with irregular cycle lengths and durations. **(A)** Voltage time series including unused pre-training data (gray), training data (blue), and testing (prediction) data (black). **(B)** Corresponding APDs, following the same color scheme as **(A)**. **(C)** Zoomed in voltage trace corresponding to the shaded region in **(A,B)** showing the irregular action potential shapes and durations.

The third dataset consists of irregular activity recorded from zebrafish hearts subjected to constant diastolic interval (DI) pacing (Cherry, 2017; Zlochiver et al., 2017); see Figure 3. All experimental procedures were approved by the office of Research Integrity Assurance of Georgia Tech under IACUC A100416. Zebrafish (*Danio rerio*) of either sex were anesthetized via cold water bath. Following anesthesia, hearts were quickly excised and immersed in Tyrode's solution (in mM: NaCl 124, KCl 4, NaHCO_3_ 24, NaH_2_PO_4_·H_2_O 0.9, MgCl_2_·6H_2_O 2, dextrose 5.5). Blebbistatin, used to stop contraction without major effects on electrophysiology (Fenton et al., 2008; Kappadan et al., 2020), was added to Tyrode's solution 20–30 min prior to data acquisition to help suppress heart motion. The heart was held in place by insect pins which attached the bulbus arteriosus to the bottom of a Sylgard-lined Petri dish. Stimulation was applied through AgCl bipolar electrodes placed on opposite sides of the heart close by to stimulate via electric field.

Intra- and extracellular voltages were acquired by two glass micropipettes containing 2.5 M KCl solution fastened into microelectrode holders (MEH3SFW, World Precision Instruments). Ag/AgCl half cells within the microelectrode holders sent signals to be buffered by pair of DC-powered preamplifiers (Electro 705, World Precision Instruments), which were connected together to output a differential measurement of transmembrane voltage. This transmembrane voltage was then split into two paths. One path led through a BNC breakout board (BNC-2110, National Instruments) to be read by a DAQ (PCIe-6341, National Instruments) and written to a file at 10,000 samples/s by a computer running a custom-built MATLAB script. Transmembrane voltage was also sent through a custom-built circuit that applied a gain and offset to the signals before being read by an Arduino Due. The Arduino Due then interpreted signals on-the-fly and determined when the heart should be stimulated to enforce a user-set DI, which was communicated to a current source stimulus isolator (Isostim A320, World Precision Instruments) that stimulated the heart.

Although constant-DI pacing can lead to stable and predictable APDs (Kulkarni et al., 2018), in our recordings APDs were highly variable despite the constant DI maintained. This high variability may result from the much smaller DIs used compared to the values used by Kulkarni et al. (2018), which were very close to the alternans bifurcation period. Figure 3 shows the experimental dataset voltage trace and APDs, including over 100 training (blue) and over 20 testing (black) action potentials. Stimulus artifacts were removed using spline interpolation in a pre-processing step.

### 2.3. Implementation Details

All methods were implemented in MATLAB (R2020b) and were run on the same computer equipped with a 1.4 GHz Quad-Core Intel Core i5 processor and 8 GB of RAM, operating with macOS Big Sur (Version 11.4).

#### 2.3.1. Hyperparameter Selection

The optimum values of various hyperparameters required for each method were tuned through an extensive grid search, the set of values for which are given in Supplementary Table 1. The ranges of the hyperparameter values used for the grid search and the number of values tested were chosen according to the results of initial experiments to generate reasonable results and also factor in the observed level of sensitivity of the employed approaches to each hyperparameter. Optimal hyperparameter values were obtained for each network size and dataset; see Supplementary Tables 2–6. Therefore, the results presented reflect the best attainable performance of each method for a given network size for each dataset.

#### 2.3.2. Gated RNN Implementations

The LSTM and GRU networks are constructed using the MATLAB Deep Learning toolbox, where the network topologies are specified by a graph of layers. To predict the action potential time series multiple steps into the future, a sequence-to-sequence regression LSTM architecture is employed that entails several main components. First, a sequential input layer is required to feed the input time series into the network. Then, an LSTM layer is used to learn the long-term dependencies between the time steps of the input sequential data. Finally, a fully connected layer connects the LSTM layer to a regression output layer to complete the design. The architecture of the GRU networks is the same as for the LSTM network except for employing a GRU layer instead of an LSTM layer. In addition to the single-layer architectures, multi-layer networks with multiple stacked gated layers are also tested in this work.

The main hyperparameters to configure in gated RNNs include the number of hidden layers and hidden units, the optimizer for the training network, and the hyperparameters related to the optimization solver, such as the maximum number of epochs, learning rate, learning rate drop factor, and regularization factor. Due to the high computational costs of gated RNNs, running an exhaustive grid search on all hyperparameters is not pragmatically feasible. Therefore, based on our initial experiments, some of these hyperparameters are set while the grid search determines the optimum values of those demonstrating a more significant role in the performance of the network. Accordingly, the Adam optimizer (Kingma and Ba, 2014) is employed for training the network with the MATLAB default training configurations and the maximum number of 30 epochs. Then, the grid search is employed to determine the optimum number of hidden layers and the initial learning rate (Supplementary Tables 2, 3).

The trained network then can be used to predict the response of the system for the next time step. To forecast voltage values multiple steps ahead, a recursive approach is adopted in which at each time step, the response is predicted using the trained network and the network state is updated correspondingly. This predicted value is featured as the input for the next time step prediction. This procedure is repeated to predict the voltage response for the entire prediction horizon.

#### 2.3.3. Echo State Network Implementations

The baseline ESN technique is implemented based on the original tutorial presented by Jaeger (2002) and the practical guide presented by Lukoševičius (2012). The reservoir graph is generated using the Erdős–Rényi algorithm (Bollobás, 2001), after which it is rescaled and updated to satisfy the echo state property of the network (Yildiz et al., 2012) ensuring that the effect of initial conditions should vanish progressively and the reservoir state should asymptotically depend only on the input signals. The same procedure is conducted to construct the reservoirs in the clustered and hybrid ESNs. More specifically, in the hybrid ESN, the reservoir graph is generated with the same randomized approach, while in the clustered ESN, the sub-reservoir clusters are generated first, then connected to each other randomly, where an additional hyperparameter specifies the probability of the inter-cluster connections.

During the training of an ESN, the first initial steps of the network states are discarded to wash out the initial states and ensure the network dynamics are fully developed. Here, the first ten beats are considered as the transient phase and their corresponding state values are not used for training the networks.

Compared to the gated RNNs, the number of hyperparameters that play a more significant role in network performance in RC techniques is considerably higher, and the performance of the network highly depends on finding a good set of hyperparameters, including the number of neurons in the reservoir, connection probability used in the Erdős–Rényi graph generation step, reservoir spectral radius, input weight scale, leaking rate, and ridge regression regularization factor. Additionally, the number of clusters and the knowledge-based model are additional parameters to consider in clustered and hybrid ESNs, respectively. Although the size of the hyperparameter grid search space grows exponentially in RC techniques and is much higher compared to that of gated RNNs, because of the lower computational efforts required in ESN approaches, we obtained grid search results two times faster than for the gated RNNs.

Note that due to the random nature of ESNs and the intrinsic sensitivity of the network to the initial values of the parameters, the results for each network size are averaged over 10 experiments with different seed values for the random number generator.

#### 2.3.4. Data Resampling

Although it is common to obtain data at a particular fixed time resolution, such a resolution often is not optimal; for example, it may contain so many points that it is difficult to obtain a good fit. More generally, an imbalanced distribution of data points in a time series can significantly deteriorate the performance of time-series prediction techniques. In such situations, a certain range of values are overrepresented compared to the rest of the time series, giving rise to a bias toward the values or behaviors that occur more frequently in the sequence. For example, in the case of an action potential time series, sampling that is uniform in time causes the upstroke phase, associated with the rapid depolarization of the cell membrane potential, to be underrepresented compared to the rest of the time series. Therefore, it is expected that prediction techniques may fail to correctly capture the upstroke phase in such cases and thus may produce a poor forecast overall. A common approach for tackling such issues is the use of resampling strategies (Moniz et al., 2017), which operate on the training dataset to make the distribution of the data points more balanced in terms of their information content.

In this work, we implement an under-sampling technique in which each data point is only included in the dataset if its voltage is sufficiently distinct from the last included data point, thereby ensuring more data points where the voltage changes rapidly. In this approach, two consecutive data points are considered distinct if the difference between the voltage values is greater than or equal to a threshold. If the threshold is set to zero, the dataset remains the same. In contrast, a very large threshold will result in great information loss and important features will be removed from the action potential time series. Therefore, the resampling threshold is also treated as a hyperparameter so that its optimum value is determined along with the other hyperparameters by the grid search. We also include a data point if the time since the last included data point exceeds a separate threshold, which is also treated as a hyperparameter, to ensure there is a sufficient density of points in portions of the action potential where the voltage changes slowly. Supplementary Table 1 illustrates the possible values of the resampling thresholds used in the grid search. We found a significant increase in the predictive accuracy when using this resampling strategy and it is used for all results shown here.

#### 2.3.5. Univariate vs. Multivariate Time Series Prediction

In practice, the action potential forecasting task entails predicting one variable (voltage) over time, resulting in a univariate time series. However, the input time series can be either a univariate or multivariate time series. The former occurs when the input data is assumed to be endogenous and is not driven by an external stimulus. The latter portrays cases in which cardiac cells are stimulated exogenously; in such a case, the pacing stimulus can also be introduced to the network along with the cardiac voltage signal. In this work, both scenarios are considered. Accordingly, the univariate input models are employed for forecasting the Noble dataset, where the auto-oscillatory nature of this model eliminates the requirements of applying an external stimulus. In contrast, both the FK dataset, which uses random stimulus timings, and the experimental dataset, which uses varying stimulus timings owing to the constant DIs but variable APDs, are used with multivariate time series prediction, which incorporates the pacing stimulus signal. In the case of the experimental data, the timing of applied stimuli is not directly available; thus, a pre-processing step is applied to detect the starting point of each beat in time and then a 2-ms stimulus current with a relative magnitude of 0.2 is used to generate the pacing stimulus signal. This process generates a stimulus current that is then resampled so that stimulus values are available for each resampled voltage data point. Our initial experiments demonstrate that the magnitude of the stimulus does not affect the quality of the predictions in this setup, but introducing the stimulus signal considerably improves the predictive ability in the first place.

Supplementary Figures 2A,C illustrate the architectures of the baseline and clustered ESNs, respectively, that are used for the univariate input case. To accommodate the pacing stimulus signal in multivariate input settings, these architectures are updated to include one more feature in the input layer (Supplementary Figures 2B,D). Introducing the stimulus information into the hybrid ESN approach is inevitable because in this architecture, the knowledge-based model should be synchronized with the input action potential time series to operate simultaneously. Therefore, the hybrid ESN is essentially developed for a multivariate input case (Supplementary Figure 2E). Accordingly, the pre-processing described step can be employed to extract the timing of the stimulus current. Similarly, in gated RNNs, the multivariate input case can be handled by adjusting the number of inputs in the sequential input layer.

#### 2.3.6. Evaluation Metrics

To assess prediction accuracy, we use the root mean square error (RMSE) metric:


(6) $$\begin{array}{l} RMSE=\sqrt{\frac{1}{n}\overset{n}{\underset{i=1}{\sum }}{\left({\hat{V}}_{i}-V_{i}\right)}^{2}}. \end{array}$$

where *V*_*i*_ and ${\hat{V}}_{i}$ are the target and predicted outputs, respectively, and *n* denotes the length of the test dataset. Note that the voltage values of the Noble and experimental datasets have been linearly rescaled to be between zero and one. The FK model is already scaled so that its upstroke reaches a maximum of one; no further rescaling is performed. As discussed in section 2.3.4, in all cases, the dataset values used here are not uniformly spaced in time.

We also assess error by comparing action potential durations (APDs). We define an APD as the time interval over which the voltage during an action potential is continuously larger than the threshold value, which is selected as 0.3 for the synthetic datasets (FK and noble) and 0.35 for the experimental dataset.

## 3. Results

### 3.1. FK Model-Derived Dataset

**Figure 4:**
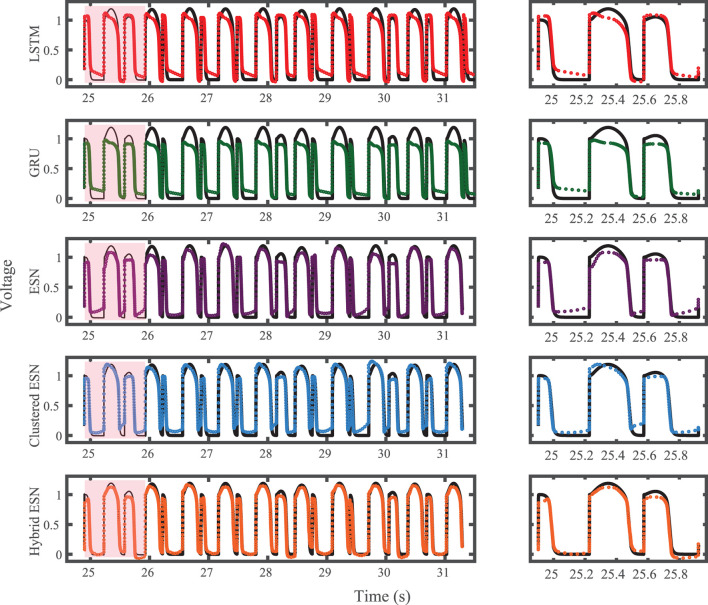
FK dataset action potential prediction results obtained for the five methods using a fixed network size of 100 neurons. Test data are shown in black for reference and the predictions in color. **(Left)** All predicted APs. **(Right)** Zoomed view of the first three predicted APs.

**Figure 5:**
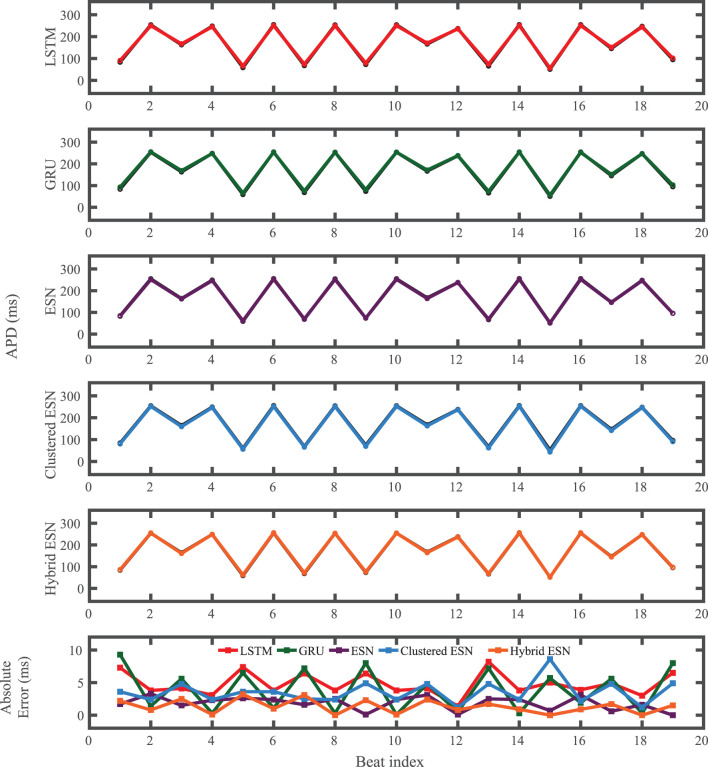
FK dataset APD prediction results obtained for the five methods using a fixed network size of 100 neurons. APDs from data used for testing are shown in black for reference and predicted APDs are shown in color. Absolute error in APD prediction is shown in the bottom subplot, with color corresponding to prediction method.

The FK model-derived dataset (hereafter referred to as the FK dataset), shown previously in Figure 1, includes irregularity in action potential shapes and durations through the use of cycle lengths drawn from a normal distribution. Figure 4 shows the 19 action potentials predicted by the five methods (LSTM, GRU, ESN, clustered ESN, and hybrid ESN) for a fixed network size of 100 hidden units. All five methods match the action potential upstrokes and downstrokes well. As a result, the predictions for APD by all methods have very low error (below 10 ms) across all 19 beats with no growth over time, as shown in detail in Figure 5, despite the irregular alternans present in the dataset. However, different methods exhibit different prediction accuracies for the plateau and rest phases of the action potentials. Specifically, the hybrid ESN does the best job of matching voltage values during these phases; the ESN approaches produce good results during the plateau but show depolarization preceding each action potential rather than remaining at a stable rest potential. The LSTM and GRU methods show the largest discrepancies, including plateau height mismatches and significant slowing in repolarization leading to elevated resting potentials.

**Figure 6:**
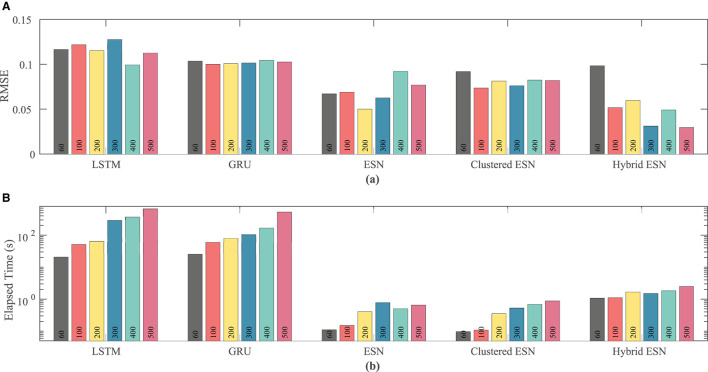
Comparison of RMSE **(A)** and computational time **(B)** for each method and network size tested for the FK dataset.

Network size has a limited effect on overall accuracy as measured by RMSE. Figure 6 shows that there is no clear trend in error as the network size is increased, except that the hybrid ESN error is much lower for networks with at least 100 neurons. The hybrid ESN also has the lowest or near lowest error for all cases with at least 100 neurons, while the ESN and clustered ESN methods generally achieve slightly lower error than the LSTM and GRU methods.

The lower plot in Figure 6 demonstrates that for a fixed network size the ESN and clustered ESNs accomplish the combination of training and prediction tasks much faster—by roughly two orders of magnitude—than the LSTM and GRU methods, with the hybrid ESN placing in between. These timing differences across the methods are maintained across all network sizes. However, within each individual method, the time for training and prediction increases with the number of neurons, except for the hybrid ESN method, for which such a trend is less clear.

### 3.2. Noble Model-Derived Dataset

**Figure 7:**
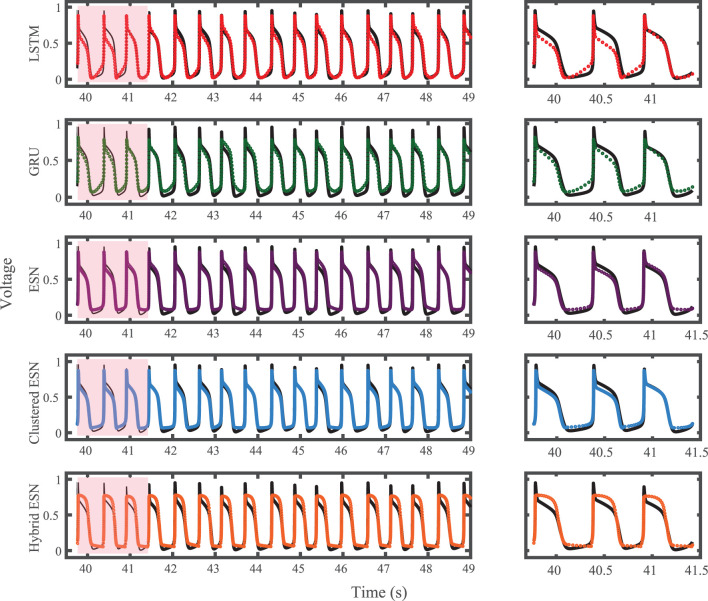
Noble dataset action potential prediction results obtained for the five methods using a fixed network size of 100 neurons. Test data are shown in black for reference and the predictions in color. **(Left)** All predicted APs. **(Right)** Zoomed view of the first three predicted APs.

**Figure 8:**
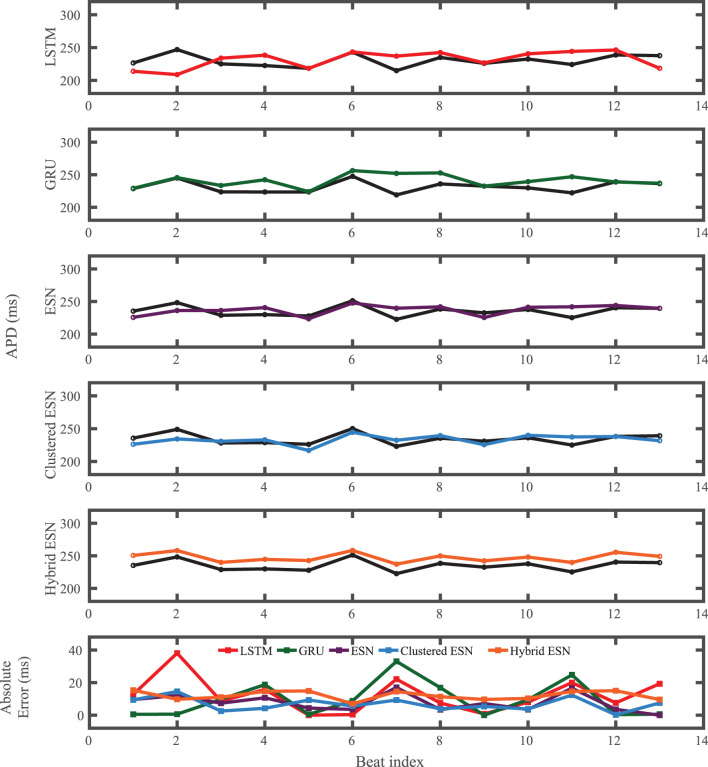
Noble dataset APD prediction results obtained for the five methods using a fixed network size of 100 neurons. APDs from data used for testing are shown in black for reference and predicted APDs are shown in color. Absolute error in APD prediction is shown in the bottom subplot, with color corresponding to prediction method.

The results of using the five methods to predict action potentials in the Noble dataset (shown in Figure 2) using a fixed network size of 100 can be seen in Figure 7, which shows the voltage traces, and in Figure 8, which shows the predicted APDs and absolute error in APD. The LSTM predictions generally achieve good agreement throughout the testing phase, with some discrepancies during the plateau and an overestimation of phase 4 depolarization. The GRU predictions are similar except that they repolarize less completely and consistently underestimate the peak upstroke voltage. The ESN and clustered ESN methods show improved accuracy with relatively minor discrepancies. In contrast, the hybrid ESN exhibits a very different action potential shape more in line with the capabilities of the knowledge-based model and consistently overestimates APD.

**Figure 9:**
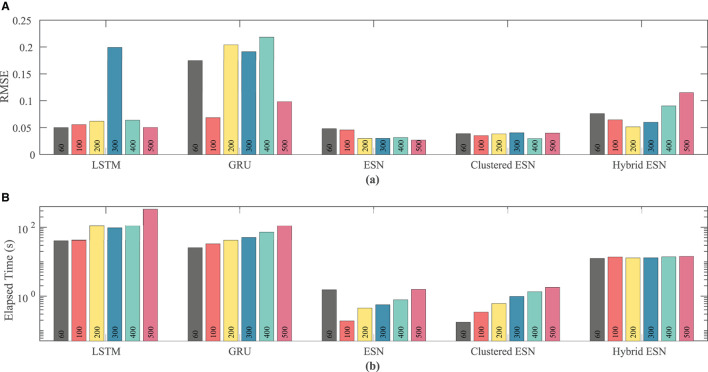
Comparison of RMSE **(A)** and computational time **(B)** for each method and network size tested for the Noble dataset.

Figure 9 shows that the ESN and clustered ESNs achieve the lowest RMSE across different network sizes. The hybrid ESN and LSTM methods perform relatively well across most network sizes but produce larger RMSE values for some network sizes. GRUs have the highest error for most network sizes for this dataset. It is difficult to discern a clear trend in accuracy with increased network size; prediction method differences appear to have stronger effects.

As with the FK model, the RNN approaches consistently take longer than the ESN and clustered ESN approaches, with the hybrid ESN in between. For network sizes of at least 100 hidden units, the ESN and clustered ESN methods require about two orders of magnitude less computational time than the RNN methods. For all approaches, there is a modest increase in computational time for 100 or more hidden units as the network size increases, with the exception of the hybrid ESN, for which the computational time is approximately constant across all network sizes tested.

### 3.3. Experimental Dataset

**Figure 10:**
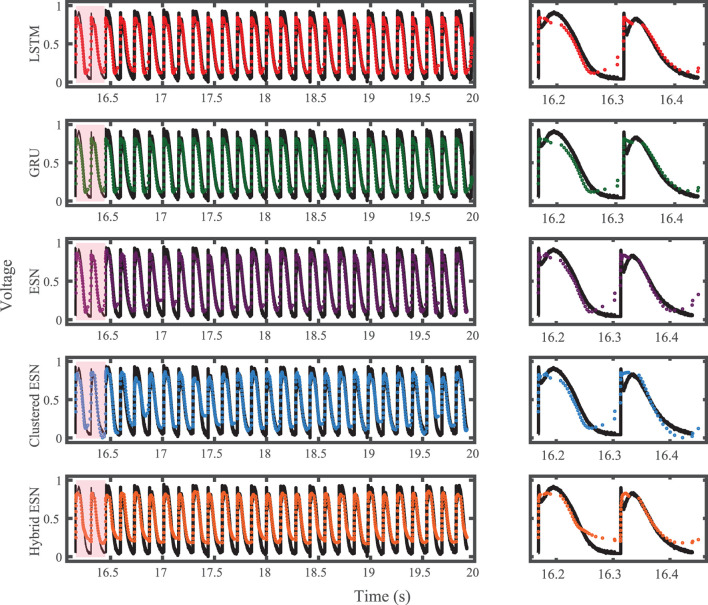
Experimental dataset action potential prediction results obtained for the five methods using a fixed network size of 100 neurons. Test data are shown in black for reference and the predictions in color. **(Left)** All predicted APs. **(Right)** Zoomed view of the first two predicted APs.

**Figure 11:**
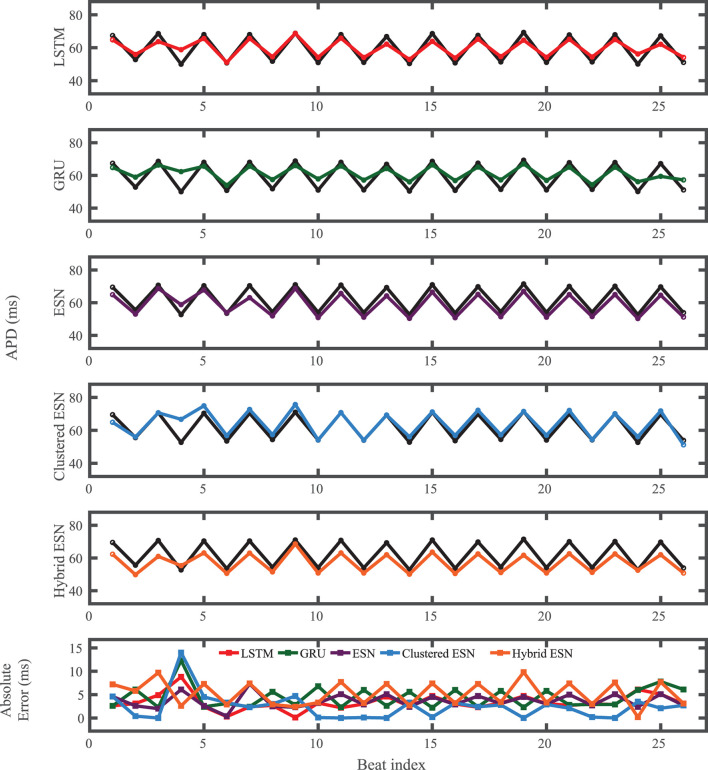
Experimental dataset APD prediction results obtained for the five methods using a fixed network size of 100 neurons. APDs from data used for testing are shown in black for reference and predicted APDs are shown in color. Absolute error in APD prediction is shown in the bottom subplot, with color corresponding to prediction method.

For the experimental dataset, obtained from zebrafish paced using a constant-DI protocol and shown in Figure 3, all five methods are able to reconstruct most of the action potential features, as demonstrated in Figure 10 with 100 hidden units. With each method, the discrepancies in predicted voltage values occur mostly during the portions of the action potentials with smaller voltage derivatives, the plateau and the rest phase. All methods underestimate the plateau height and fall short of repolarizing fully, with the hybrid ESN continuing to repolarize slowly throughout what should be the rest phase while the other methods produce depolarization during this phase. The performance does not vary significantly over the full prediction series, although some individual action potentials are not fit well. As shown in Figure 11, the largest APD differences from the true values occur for the hybrid ESN, with the clustered ESN yielding especially good results. The ESN and hybrid ESN consistently underestimate APD values, and the APD values predicted by the LSTM and GRU methods are generally less extreme than the true APDs (that is, the long APDs are predicted to be shorter and the short APDs are predicted to be longer).

**Figure 12:**
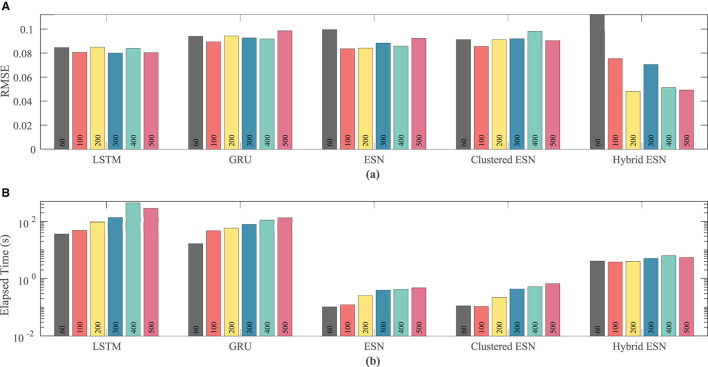
Comparison of RMSE **(A)** and computational time **(B)** for each method and network size tested for the experimental dataset.

As the number of neurons is changed, there is no clear effect on accuracy, except possibly for the hybrid ESN, which appears to have a slight trend toward lower RMSE with more neurons; see Figure 12. In contrast, the time required for training and prediction shows the same trend as for the other data sets, with the LSTM and GRU approaches requiring about two orders of magnitude more time for the same network size than the ESN and clustered ESN approaches, and the hybrid ESN in between. All the methods show a trend toward increasing time with increasing network size, except for the hybrid ESN, which as before appears insensitive to network size.

## 4. Discussion

In this paper, we tested five different ML time series prediction methods, two based on RNNs and three based on a type of reservoir computing, to predict irregular voltage dynamics arising from random or chaotic effects for three cardiac datasets. We found that for clinically relevant intervals (1–3 s, or 6–19 beats at 160 ms) for the detection of cardiac arrhythmia in embedded devices (Madhavan and Friedman, 2013), we were able to predict voltage traces that closely match the true dynamics. We showed that for all datasets considered, all five prediction methods could produce accurate forecasts of both voltage and APD for around 15–20 action potentials (as long as was tested). With the exception of GRU predictions for the Noble dataset, RMSE values on the order of 0.1 normalized voltage units or lower could be attained for every combination of dataset and prediction method. APD errors were typically less than 5 ms for both the FK and experimental datasets, with larger typical errors of around 20 ms (sometimes more) for the Noble dataset. Over the measured interval, none of the methods exhibited any long-term trend in the APD error, indicating that the methods have seen sufficient training data to accurately model the real action potential response to stimulation and the associated APD distribution.

In addition, we demonstrated that the ESN approaches achieved lower error than the RNN approaches for the synthetic datasets and that the hybrid ESN achieved the best accuracy for the experimental dataset. Furthermore, the accuracy obtained, as measured by RMSE, was largely independent of network size. The time required for training and prediction typically was about two orders of magnitude lower for the ESN and clustered ESN architectures compared to the LSTM and GRU approaches, with the hybrid ESN timing in between. Computation time also generally grew with the network size, with the exception of the hybrid ESN, where computational time was essentially constant across all network sizes considered. We expect this insensitivity occurred because the time associated with solving the knowledge-based model (essentially a set of differential equations describing a cardiac cell), which is independent of network size, was more expensive than the cost of the ESN itself and thus dominated the total training and prediction time.

We investigated different types of dynamics, including those influenced by underlying randomness and chaos. We found the lowest RMSE values for the FK and experimental datasets and the highest RMSE values for the chaotically-driven Noble dataset. For the FK dataset, the hybrid ESN typically achieved the lowest error for all but the smallest network size, with the other ESN approaches achieving slightly less error than the LSTM and GRU methods. The Noble dataset elicited a particularly poor performance for the GRU method, with RMSE values typically three or four times larger than for the other methods; also, the hybrid ESN did not perform as well as the other ESNs. However, it is possible that use of a univariate time series in this case contributed to lower accuracy, rather than just the chaotic dynamics alone. For the experimental dataset, which likely has elements of both randomness and chaos, the hybrid ESN generally achieved the lowest error, with the other methods producing similar RMSE values. Overall, our results indicate that the ESN architecture provides better performance than LSTM and GRU approaches for the voltage forecasting task.

### 4.1. Effects of Algorithmic Choices

The action potential time series used in this work were highly imbalanced. In this study, although we found the need to downsample the original data for use with testing and training, we did not perform systematic studies regarding how to optimize this task. As a general observation, starting from the initial highly imbalanced time series, by increasing the sampling spacing and reducing the number of data points, the training and testing errors were reduced. However, the information loss caused by removing data points is the obvious side effect if the spacing becomes too large. Our choice of tying the time spacing to changes in voltage ensured good resolution during rapidly changing parts of action potentials, including the upstroke, but led to a lack of points during the rest and plateau phases, contributing to apparent errors during these times of slow changes in voltage. Further studies are required to investigate various spacing and resampling strategies to propose an optimal approach.

Although our results illustrate that ESNs provide the best prediction accuracy together with the lowest computational times in most cases for the methods and datasets considered, the ESN approach shows the most sensitivity to the hyperparameter and network parameter values. Our grid search results demonstrated a wide variability in the prediction performance obtained by various ESNs with very similar configurations. This motivates more study to improve the robustness of this approach. Among the three RC techniques used in this work, the hybrid ESN showed the least sensitivity to the hyperparameter values. We expect that the knowledge-based model promotes the predictive ability of the network by generating an approximate action potential, which the network perturbs to resolve the precise AP shape.

Incorporating the pacing stimulus into a multivariate input setup considerably improved the prediction performance of the network over using a univariate voltage input and extended the forecasting horizon to a higher number of beats. In the absence of the stimulus information, depending on the dynamics of the system, the predictions remain accurate only for the first few beats after the training.

### 4.2. Limitations and Future Work

Our study contains a number of limitations. First, we studied a limited number of datasets. It is possible that different types of dynamics (e.g., more strongly chaotic) could lead to different results, and in particular experimental data from other sources could prove more difficult to predict. In addition, we did not study how much training data was needed to obtain good results. Furthermore, it remains an important open question how long the action potential predictions will remain accurate without deteriorating, although in this case we have found lower bounds.

We also considered a small number of time series prediction methods. There are many variations on these methods (Chandra et al., 2021; Han et al., 2021) and it is possible that performance improvements could be achieved. Even choosing different settings for the methods considered, such as a different number of clusters for the clustered ESNs, potentially could affect performance. There are also different types of prediction methods that we did not consider. For example, ESNs have been connected to vector autoregression (VAR) (Bollt, 2021), thereby motivating additional studies of VAR for prediction. It also would be interesting to study the accuracy of predictions of APD obtained by training on APD values only.

For the hybrid ESN, we only considered the use of one knowledge-based model, the Corrado-Niederer update of the Mitchell-Schaeffer model. It is possible that different model choices could affect the accuracy or computational time of the hybrid method; for example, an even simpler two-variable model like the FitzHugh-Nagumo model could potentially make the hybrid ESN approach more competitive with the other ESNs considered here, while a knowledge-based model that is matched to the data-generating model might present a near-trivial prediction task. Additionally, more complex cardiac cell models with detailed calcium dynamics may have an impact on long-term tissue memory. In practice, this long-term change in the cardiac cell may lessen the predictive power of the presented ML models over long time intervals.

We also note that there is a close connection between ML-based methods and data assimilation. In the cardiac case, Kalman filter-based methods including data assimilation have been used thus far for reconstruction (Muñoz and Otani, 2010, 2013; Hoffman et al., 2016; Hoffman and Cherry, 2020; Marcotte et al., 2021), but they also can be used for forecasting, as is more typical in data assimilation's original weather forecasting context (Hunt et al., 2007). It may be beneficial to pursue approaches that seek to merge data assimilation and machine learning for this task (Albers et al., 2018; Brajard et al., 2020; Gottwald and Reich, 2021).

Along with extensions of our present work to address the issues discussed above, in the future we intend to consider predicting cardiac voltage dynamics during the development of arrhythmias. We expect this goal may necessitate the use of spatially extended models of cardiac tissue as part of the prediction process, although handling the information from spatial neighbors requires very large networks that will pose new computational challenges. The combination of ESNs and local states (Pathak et al., 2018; Zimmermann and Parlitz, 2018) or specialized deep-learning architectures (Herzog et al., 2018) may be useful in tackling such problems, but these methods remain computationally demanding and may require new approaches. In addition, we may need to carefully consider the types of dynamics included in the training data in order to accurately predict transitions between different types of dynamics, such as the transition from normal rhythm to tachycardia or the transition from tachycardia to fibrillation. Accurate prediction of such transitions may lead to advances in control designed to prevent the development of fatal arrhythmias.

## Data Availability Statement

The raw data supporting the conclusions of this article will be made available by the authors, without undue reservation.

## Ethics Statement

The animal study was reviewed and approved by the Office of Research Integrity Assurance of Georgia Tech under IACUC A100416.

## Author Contributions

SS, FF, YS, and EC contributed to developing the study concept. SS, CM, and EC contributed to study design. SS and EC developed the synthetic datasets and analyzed and data. CH and FF obtained the experimental datasets. SS, FF, and EC wrote the manuscript. CM, CH, and YS critically revised the manuscript. All authors contributed to the article and approved the submitted version.

## Funding

This study was supported in part by the National Science Foundation grants CMMI-2011280 (EC) and CMMI-1762553 (FF) and by the National Institutes of Health grant 1R01HL143450-01 (EC and FF).

## Conflict of Interest

The authors declare that the research was conducted in the absence of any commercial or financial relationships that could be construed as a potential conflict of interest.

## Publisher's Note

All claims expressed in this article are solely those of the authors and do not necessarily represent those of their affiliated organizations, or those of the publisher, the editors and the reviewers. Any product that may be evaluated in this article, or claim that may be made by its manufacturer, is not guaranteed or endorsed by the publisher.
