# Supplementary material for: Long-Time Prediction of Arrhythmic Cardiac Action Potentials Using Recurrent Neural Networks and Reservoir Computing
Source: Front Physiol. 2021 Sep 27;12:734178. doi: 10.3389/fphys.2021.734178 (PMC8502981; doi:10.3389/fphys.2021.734178)
Supplement: Supplementary file 1 [file Data_Sheet_1.pdf]

Supplementary Material

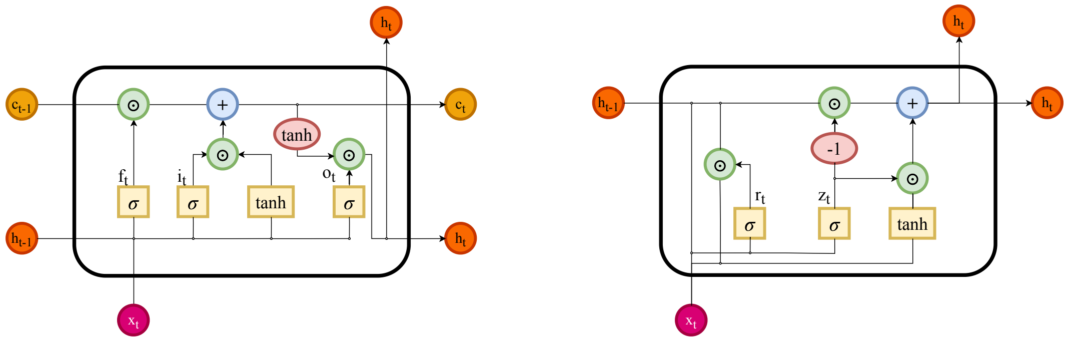

(a) **(b)**

Figure S1. Architectures for the recurrent neural network approaches. **(a)** Long-short term memory network components. **(b)** Gated recurrent unit components.

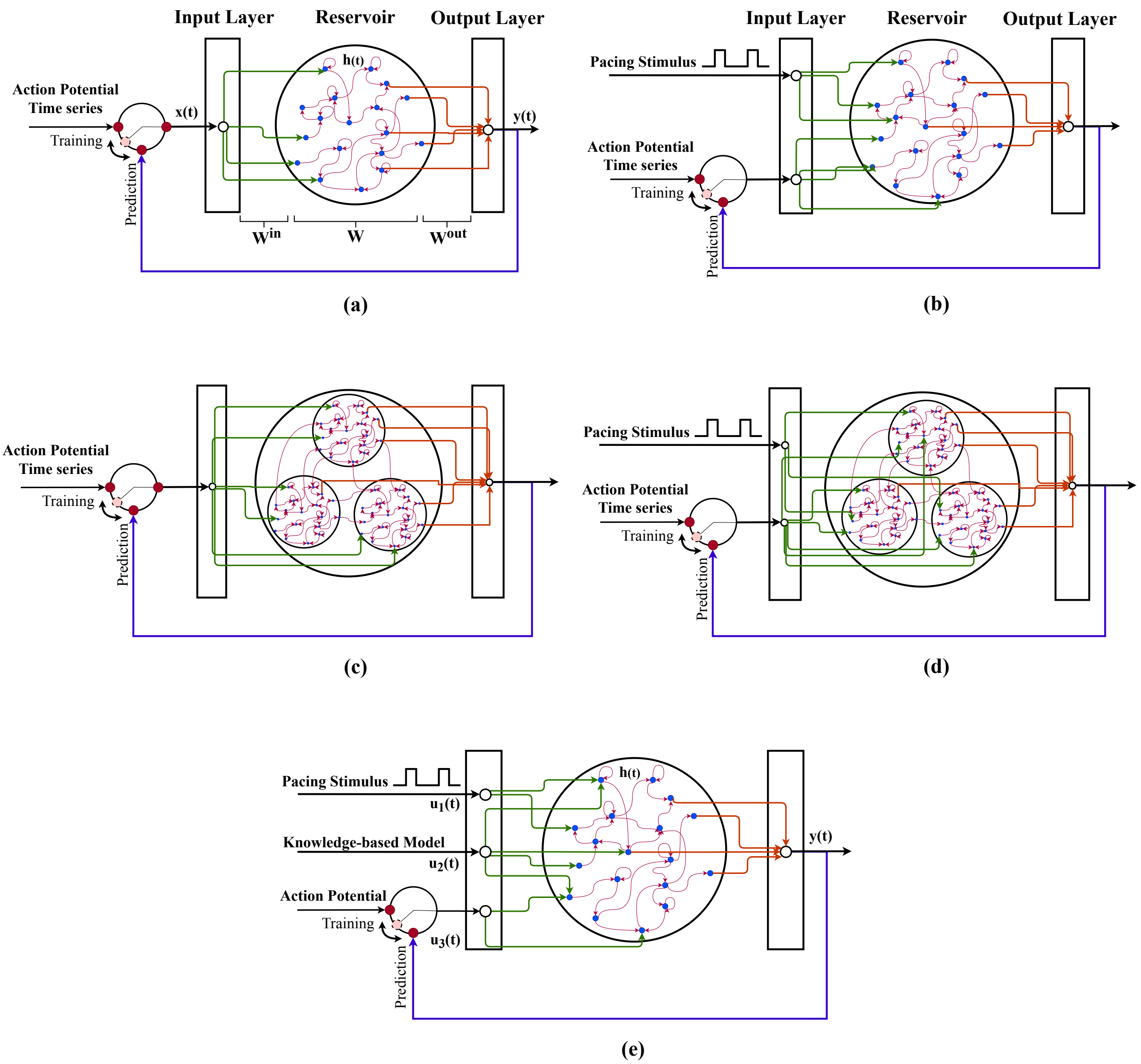

Figure S2. Architectures for the reservoir computing approaches. **(a)** ESN components for univariate time series. **(b)** ESN components for multivariate time series including stimulus information. **(c)** Clustered ESN components for univariate time series. **(d)** Clustered ESN components for multivariate time series including stimulus information. **(e)** Hybrid ESN components for multivariate time series including stimulus information.

.

Table S1. Hyperparameter values used for the grid search optimization for each prediction method. The resampling voltage threshold δ defines the minimum difference between the voltage values of each two consecutive data points, which is used as the first criterion for resampling the action potential time series. The resampling time threshold τ determines the maximum time gap (in ms) between each two consecutive data points. The learning rate η is the initial learning rate used by the Adam optimizer for training the gated RNNs. Input weight scales σ^1_{in}, σ^2_{in}, and σ^3_{in} are the scalars that are multiplied by the corresponding columns of the input weight matrix in the ESNs to adjust the magnitude of the input signals including the action potential, the pacing stimulus, and the knowledge-based model, if applicable. The reservoir weight matrix is also scaled such that its spectral radius, defined as the largest among the absolute values of the eigenvalues, is equal to the selected spectral radius ρ. The leaking rate α determines the amount of excitation discarded by the leaky integrator neurons and used to control the rate of the reservoir update dynamics discretized in time. The regularization parameter λ determines the ridge regression regularization factor employed for calculation of the readout weights in ESNs. The connection probability pr is the probability of having an edge between each two neurons in the reservoir, which controls the sparsity of the reservoir graph. The inter-cluster probability pr_c is the probability of having an edge between each two nodes from different sub-reservoirs in the clustered ESN approach.

Methods	Parameters	Values
	Resampling voltage threshold (δ)	$\{0.00, 0.01, 0.02, 0.03, 0.04\}$
LSTM	Resampling time threshold (τ)	$\{20, 30, 40, 50\}$
	Number of layers	$\{1, 2, 4\}$
	Learning rate (η)	$\{0.001, 0.002, 0.005, 0.010, 0.150\}$
	Resampling voltage threshold (δ)	$\{0.00, 0.01, 0.02, 0.03, 0.04\}$
GRU	Resampling time threshold (τ)	$\{20, 30, 40, 50\}$
	Number of layers	$\{1, 2, 4\}$
	Learning rate (η)	$\{0.001, 0.002, 0.005, 0.010, 0.150\}$
	Resampling voltage threshold (δ)	$\{0.00, 0.01, 0.02, 0.03, 0.04\}$
	Resampling time threshold (τ)	$\{20, 30, 40, 50\}$
	Input weight scale (action potential, σ^1_{in})	$\{0.02, 0.05, 0.10, 0.20, 0.50, 0.80\}$
ESN	Input weight scale (pacing stimulus , σ^2_{in})	$\{0.02, 0.05, 0.10, 0.20, 0.50, 0.80\}$
	Spectral radius (ρ)	$\{0.80, 0.85, 0.90, 0.99, 1.05, 1.25, 1.50\}$
	Leaking rate (α)	$\{0.20, 0.30, 0.40, 0.50, 0.60, 0.70, 0.80, 0.90, 1.00\}$
	Regularization (λ)	$\{10^{-7}, 10^{-6}, 10^{-5}, 10^{-4}, 10^{-3}, 10^{-2}\}$
	Connection probability (pr)	$\{0.01, 0.02, 0.05, 0.10, 0.15, 0.20\}$
	Resampling voltage threshold (δ)	$\{0.00, 0.01, 0.02, 0.03, 0.04\}$
	Resampling time threshold (τ)	$\{20, 30, 40, 50\}$
	Input weight scale (action potential, σ^1_{in})	$\{0.02, 0.05, 0.10, 0.20, 0.50, 0.80\}$
	Input weight scale (pacing stimulus , σ^2_{in})	$\{0.02, 0.05, 0.10, 0.20, 0.50, 0.80\}$
Clustered ESN	Number of clusters (n_c)	$\{2, 3, 4, 5\}$
	Spectral radius (ρ)	$\{0.80, 0.85, 0.90, 0.99, 1.05, 1.25, 1.50\}$
	Leaking rate (α)	$\{0.20, 0.30, 0.40, 0.50, 0.60, 0.70, 0.80, 0.90, 1.00\}$
	Regularization (λ)	$\{10^{-7}, 10^{-6}, 10^{-5}, 10^{-4}, 10^{-3}, 10^{-2}\}$
	Intra-cluster connection probability (pr)	$\{0.60, 0.7, 0.80, 0.85, 0.90, 0.95, 0.98\}$
	Inter-cluster connection probability (pr_c)	$\{0.01, 0.02, 0.05, 0.10, 0.15, 0.20\}$
	Resampling voltage threshold (δ)	$\{0.00, 0.01, 0.02, 0.03, 0.04\}$
	Resampling time threshold (τ)	$\{20, 30, 40, 50\}$
	Input weight scale (action potential, σ^1_{in})	$\{0.02, 0.05, 0.10, 0.20, 0.50, 0.80\}$
	Input weight scale (pacing stimulus , σ^2_{in})	$\{0.02, 0.05, 0.10, 0.20, 0.50, 0.80\}$
Hybrid ESN	Input weight scale (knowledge based model, σ^3_{in})	$\{0.02, 0.05, 0.10, 0.20, 0.50, 0.80\}$
	Spectral radius (ρ)	$\{0.80, 0.85, 0.90, 0.99, 1.05, 1.25, 1.50\}$
	Leaking rate (α)	$\{0.20, 0.30, 0.40, 0.50, 0.60, 0.70, 0.80, 0.90, 1.00\}$
	Regularization (λ)	$\{10^{-7}, 10^{-6}, 10^{-5}, 10^{-4}, 10^{-3}, 10^{-2}\}$
	Connection probability (pr)	$\{0.01, 0.02, 0.05, 0.10, 0.15, 0.20\}$

Table S2. Optimal hyperparameters found by grid search for the LSTM method for each dataset and network size. Hyperparameter definitions are given in Table S1.

Action potential	Network size	δ	τ	η	$layers$
Fenton-Karma	60	0.04	40	0.010	1
	100	0.04	40	0.010	1
	200	0.04	40	0.005	1
	300	0.01	30	0.002	4
	400	0.02	40	0.002	1
	500	0.01	30	0.002	4
Noble	60	0.04	40	0.002	4
	100	0.04	40	0.002	4
	200	0.02	40	0.002	4
	300	0.04	40	0.002	2
	400	0.04	40	0.010	4
	500	0.02	30	0.005	4
Experimental Data	60	0.01	40	0.005	2
	100	0.01	40	0.002	2
	200	0.01	40	0.002	2
	300	0.01	30	0.005	4
	400	0.01	40	0.002	1
	500	0.01	30	0.002	2

Table S3. Optimal hyperparameters found by grid search for the GRU method for each dataset and network size. Hyperparameter definitions are given in Table S1.

Action potential	Network size	δ	τ	η	$layers$
Fenton-Karma	60	0.04	40	0.002	1
	100	0.04	40	0.002	2
	200	0.02	40	0.002	1
	300	0.04	40	0.010	4
	400	0.04	40	0.005	4
	500	0.02	30	0.002	4
Noble	60	0.04	40	0.005	1
	100	0.04	40	0.002	4
	200	0.04	40	0.010	1
	300	0.04	40	0.002	1
	400	0.04	40	0.005	1
	500	0.04	40	0.002	4
Experimental Data	60	0.01	40	0.010	1
	100	0.01	40	0.005	4
	200	0.01	40	0.002	4
	300	0.01	40	0.002	1
	400	0.01	40	0.002	1
	500	0.01	30	0.002	1

Table S4. Optimal hyperparameters found by grid search for the ESN method for each dataset and network size. Hyperparameter definitions are given in Table S1.

Action potential	Network size	δ	τ	σ_{in}^1	σ_{in}^2	pr	ρ	α	λ
Fenton-Karma	60	0.02	40	0.02	0.10	0.05	1.05	0.70	10^{-6}
	100	0.03	40	0.10	0.10	0.05	1.05	0.80	10^{-6}
	200	0.01	40	0.02	0.10	0.10	1.05	0.80	10^{-5}
	300	0.01	40	0.02	0.10	0.05	1.05	0.80	10^{-5}
	400	0.02	30	0.10	0.10	0.02	0.85	1.00	10^{-4}
	500	0.02	40	0.02	0.10	0.02	1.05	0.70	10^{-4}
Noble	60	0.02	40	0.10	-	0.05	1.05	0.80	10^{-6}
	100	0.02	30	0.10	-	0.10	1.05	0.80	10^{-7}
	200	0.01	40	0.05	-	0.02	1.05	0.80	10^{-6}
	300	0.01	40	0.05	-	0.05	1.05	1.00	10^{-5}
	400	0.02	30	0.10	-	0.05	0.99	0.80	10^{-6}
	500	0.01	30	0.10	-	0.05	1.05	0.80	10^{-5}
Experimental Data	60	0.01	40	0.10	0.10	0.02	1.05	0.80	10^{-6}
	100	0.01	40	0.02	0.10	0.10	0.95	1.00	10^{-6}
	200	0.01	40	0.10	0.02	0.10	1.05	0.90	10^{-4}
	300	0.01	40	0.10	0.10	0.05	1.05	0.70	10^{-4}
	400	0.01	30	0.02	0.10	0.05	1.05	0.70	10^{-6}
	500	0.01	40	0.10	0.10	0.05	0.95	0.80	10^{-4}

* For the Noble dataset, stimulus information is not used so the input weight scale for the stimulus current is not applicable.

Table S5. Optimal hyperparameters found by grid search for the clustered ESN method for each dataset and network size. Hyperparameter definitions are given in Table S1.

Action potential	Network size	n_c	δ	τ	σ_{in}^1	σ_{in}^2	pr_c	pr	ρ	α	λ
Fenton-Karma	60	3	0.03	40	0.10	0.10	0.02	0.98	0.90	1.00	10^{-5}
	100	3	0.02	40	0.02	0.10	0.02	0.98	1.05	0.70	10^{-6}
	200	2	0.02	40	0.10	0.10	0.05	0.98	1.05	1.00	10^{-4}
	300	2	0.02	40	0.10	0.02	0.05	0.95	0.85	0.80	10^{-6}
	400	2	0.02	40	0.10	0.10	0.10	0.98	1.05	0.70	10^{-4}
	500	3	0.02	40	0.10	0.10	0.05	0.95	1.05	0.70	10^{-4}
Noble	60	2	0.02	40	0.10	-	0.05	0.98	0.90	0.80	10^{-7}
	100	4	0.01	30	0.10	-	0.02	0.95	0.99	0.90	10^{-7}
	200	4	0.01	40	0.10	-	0.02	0.95	0.99	0.90	10^{-7}
	300	2	0.01	40	0.10	-	0.02	0.90	0.99	0.90	10^{-7}
	400	3	0.01	40	0.10	-	0.02	0.95	0.99	0.80	10^{-7}
	500	4	0.01	40	0.10	-	0.05	0.95	0.99	0.90	10^{-7}
Experimental Data	60	3	0.01	40	0.02	0.10	0.02	0.98	1.05	0.90	10^{-5}
	100	3	0.01	40	0.10	0.02	0.02	0.98	1.05	0.70	10^{-6}
	200	3	0.01	40	0.10	0.10	0.10	0.98	1.05	0.70	10^{-6}
	300	2	0.01	40	0.10	0.02	0.10	0.98	1.05	0.80	10^{-6}
	400	3	0.01	40	0.10	0.10	0.02	0.98	1.05	0.70	10^{-5}
	500	2	0.01	40	0.10	0.10	0.10	0.95	1.05	1.00	10^{-6}

* For the Noble dataset, stimulus information is not used so the input weight scale for the stimulus current is not applicable.

Table S6. Optimal hyperparameters found by grid search for the hybrid ESN method for each dataset and network size. Hyperparameter definitions are given in Table S1.

Action potential	Network size	δ	τ	σ_{in}^1	σ_{in}^2	σ_{in}^3	pr	ρ	α	λ
Fenton-Karma	60	0.02	40	0.50	0.05	0.50	0.25	0.80	0.50	10^{-4}
	100	0.02	40	0.50	0.05	0.50	0.25	0.95	0.50	10^{-3}
	200	0.01	40	0.50	0.05	0.50	0.10	1.25	0.50	10^{-3}
	300	0.02	40	0.50	0.05	0.80	0.05	1.25	0.50	10^{-3}
	400	0.02	40	0.20	0.05	0.80	0.10	1.25	0.50	10^{-3}
	500	0.01	40	0.50	0.05	0.80	0.05	1.25	0.50	10^{-3}
Noble	60	0.02	40	0.50	0.05	0.50	0.25	0.95	0.50	10^{-3}
	100	0.02	30	0.20	0.05	0.50	0.25	0.95	0.50	10^{-3}
	200	0.02	40	0.20	0.05	0.50	0.25	0.85	0.50	10^{-4}
	300	0.02	30	0.20	0.05	0.80	0.15	0.95	0.50	10^{-3}
	400	0.02	30	0.50	0.05	0.80	0.25	0.80	0.80	10^{-4}
	500	0.02	40	0.20	0.05	0.80	0.15	0.85	0.50	10^{-3}
Experimental Data	60	0.01	40	0.20	0.02	0.50	0.15	0.99	0.80	10^{-3}
	100	0.04	30	0.50	0.02	0.80	0.15	0.99	0.50	10^{-3}
	200	0.04	40	0.50	0.02	0.50	0.10	0.99	0.50	10^{-4}
	300	0.04	30	0.50	0.02	0.50	0.15	0.95	0.50	10^{-4}
	400	0.01	20	0.20	0.02	0.50	0.10	0.99	0.50	10^{-4}
	500	0.04	30	0.50	0.02	0.80	0.15	0.99	0.80	10^{-4}
